# Morphological Characteristics of *Schistosoma mansoni* PZQ-Resistant and -Susceptible Strains Are Different in Presence of Praziquantel

**DOI:** 10.3389/fmicb.2016.00594

**Published:** 2016-04-26

**Authors:** António Pinto-Almeida, Tiago Mendes, Rosimeire Nunes de Oliveira, Sheila de Andrade Penteado Corrêa, Silmara Marques Allegretti, Silvana Belo, Ana Tomás, Fernanda de Freitas Anibal, Emanuel Carrilho, Ana Afonso

**Affiliations:** ^1^Graduate Program in Areas of Basic and Applied Biology, Instituto de Ciências Biomédicas Abel Salazar, Universidade do PortoPorto, Portugal; ^2^Medical Parasitology Unit, Global Health and Tropical Medicine, Instituto de Higiene e Medicina Tropical, Universidade Nova de LisboaLisbon, Portugal; ^3^Bioanalytical, Microfabrication, and Separations Group, Instituto de Química de São Carlos, Universidade de São PauloSão Carlos, Brazil; ^4^Departamento De Biologia Animal, Instituto de Biologia, Universidade Estadual de CampinasCampinas, Brazil; ^5^Laboratory of Parasitology, Department of Morphology and Pathology, Universidade Federal de São CarlosSão Carlos, Brazil

**Keywords:** *Schistosoma mansoni*, schistosomiasis, Praziquantel, resistance, morphology, eggs, tegument

## Abstract

Schistosomiasis is one of the most common human parasitic diseases whose socioeconomic impact is only surpassed by malaria. Praziquantel (PZQ) is the only drug commercially available for the treatment of all schistosome species causing disease in humans. However, there has been stronger evidences of PZQ-resistance on *Schistosoma mansoni* and thus it is very important to study the phenotypic characteristics associated with it. The aim of this study was to evaluate morphological alterations in *S. mansoni* PZQ-resistant adult worms and eggs, by comparing a PZQ- resistant strain obtained under PZQ drug pressure with a PZQ-susceptible strain. For this, scanning electronic microscopy was used to assess tegumental responsiveness of both strains under PZQ exposure, and optical microscopy allowed the monitoring of worms and eggs in the presence of the drug. Those assays showed that PZQ-susceptible worms exposed to the drug had more severe tegumental damages than the resistant one, which had only minor alterations. Moreover, contrary to what occurred in the susceptible strain, resistant worms were viable after PZQ exposure and gradually regaining full motility after removal of the drug. Eggs from resistant strain parasites are considerably smaller than those from susceptible strain. Our results suggest that there might be a difference in the tegument composition of the resistant strain and that worms are less responsive to PZQ. Changes observed in egg morphology might imply alterations in the biology of schistosomes associated to PZQ-resistance, which could impact on transmission and pathology of the disease. Moreover, we propose a hypothetical scenario where there is a different egg tropism of the *S. mansoni* resistant strain. This study is the first comparing two strains that only differ in their resistance characteristics, which makes it a relevant step in the search for resistance determinants.

## Introduction

Schistosomiasis is a disease caused by blood fluke trematode, *Schistosoma* spp., where *Schistosoma haematobium, Schistosoma japonicum*, and *Schistosoma mansoni* are the three main species affecting humans (Utzinger and Keiser, 2004; Gryseels et al., 2006; Colley et al., 2014). *S. mansoni* is the most widespread, being endemic in 54 countries, mostly in Africa and parts of South America (Crompton, 1999; Chitsulo et al., 2000).

Schistosomiasis is considered one of the most common human parasitic diseases whose socioeconomic impact is only surpassed by malaria among all parasitic diseases. The disease registers high rates of morbidity, in about 20 million people and mortality about 280000 deaths annually, especially in tropical and subtropical countries, namely Africa, Middle East, Caribbean, Brazil, Venezuela, Suriname, and other countries such as China, Indonesia and the Philippines [van der Werf et al., 2003; Steinmann et al., 2006; Kamel et al., 2011; World Health Organization (WHO), 2013]. It is a chronic parasitic disease which is considered a neglected disease by the World Health Organization. It has been estimated that approximately 249 million people are infected worldwide, with 780 million being at risk of infection [Steinmann et al., 2006; Caffrey, 2007; World Health Organization (WHO), 2013].

The disease transmission invariably occurs when people suffering from schistosomiasis contaminate freshwater with their excreta containing parasite eggs, which hatch in water. People become infected when larval forms of the parasite (cercariae) penetrate the skin during contact with freshwater, usually by swimming or washing (Gautret et al., 2012).

Schistosome infections can cause severe damage to various organs, mostly intestine, bladder, liver, brain and spinal cord, and cause significant morbidity, impairs childhood development and adult productivity, potentially increases susceptibility to other infections such as HIV, and, in some cases, lead to death (van der Werf et al., 2003; King and Dangerfield-Cha, 2008; Hotez and Fenwick, 2009; King, 2010; Ndeffo Mbah et al., 2013; Colley et al., 2014). Infrastructural and educational awareness can be highly effective to control schistosomiasis (Tanaka and Tsuji, 1997), however they are expensive and require levels of organization that are difficult in most developing countries (Greenberg, 2014). The use of molluscicides to eliminate intermediate host snails is another important control method, but it is also costly and often produces limited and short-term effectiveness (Sturrock, 2001) as well as having some negative environmental impact. Therefore, because there is no available vaccine or prophylaxis, current control of schistosomiasis is based only on chemotherapy using Praziquantel (PZQ) (Hotez et al., 2007).

PZQ is the only antischistosomal drug commercially available for the treatment of all human schistosome species (Cioli and Pica-Mattoccia, 2003; Fenwick et al., 2006; Doenhoff et al., 2009; Greenberg, 2014) and it presents important advantages such as mild side effects and relatively low cost (Ndeffo Mbah et al., 2013). PZQ has been available for several decades and large-scale PZQ treatment programs have produced significant reductions in both disease prevalence and intensity (Vennervald et al., 2005; Toure et al., 2008; Sesay et al., 2014). However, dependence on a single drug, which would be inadvisable for any infectious condition, is in the case of a disease with such high prevalence as schistosomiasis (Caffrey, 2007) a major concern, because it might induce the appearance of drug-resistant/tolerant parasites (Fallon et al., 1995; Gryseels et al., 2006; Doenhoff et al., 2008; Gryseels, 2012).

So far, the mechanism involved in the phenomenon of resistance to PZQ is not yet fully understood, there is only descriptions and evidences of this phenomenon *in vivo* and *in vitro* studies. For instance, Fallon and Doenhoff (1994), produced a *S. mansoni* PZQ-resistant strain in only two generations after repeated exposure to sub-lethal doses of the drug through *in vivo* artificial selection in mice. Furthermore, low cure rates in response to PZQ emerged 10–15 years ago after mass scale use in countries like Egypt and Senegal (Ismail et al., 1996; Doenhoff et al., 2002) and worms from non-cured patients were repeatedly less susceptible to PZQ when tested in mice (Cioli et al., 2004).

de Oliveira et al. (2012), evaluated the effect of PZQ on the morphology of adult *S. mansoni* susceptible to PZQ and observed that parasites exposed to the drug showed tegumental changes apparent in all male and female worms. They observed destruction of tubercles with loss of thorns and formation of vesicles around the tubercles. Since the tegument of adult *Schistosoma* is a protective sheath that plays a role in defense as well as in the uptake of nutrients, osmoregulation and excretion, damages in this structure may have major consequences to parasite viability. Thus, morphological studies are important to clarify aspects of drug-induced damage (El-Shabasy et al., 2015).

We have obtained in our laboratory by stepwise drug pressure a PZQ-resistant parasite strain (IHMT-LISBON) from a fully PZQ-susceptible parasite strain (Belo Horizonte, Brazil line). This, *S. mansoni* variant strain is 12 times more resistant to PZQ than the original susceptible one and this resistant phenotype is stable in the absence of drug pressure. This resistant parasite variant strain, obtained from infected mice, tolerates up to 1200 mg PZQ/kg of mouse body weight and is isogenic to its fully susceptible parental counterpart, except for the genetic determinants accounting for the PZQ-resistance phenotype (Pinto-Almeida et al., 2015). Besides that, data resulting from earlier study (Pinto-Almeida et al., 2015) suggested that the S. mansoni PZQ-resistant strain has different *in vitro* susceptibility to PZQ and that this difference varied greatly between male and female worms. In this context, the objective of this study was to evaluate morphological alterations (phenotypic characteristics) in the *S. mansoni* PZQ-resistance phenotype by comparing the PZQ-resistant strain obtained under PZQ drug pressure with the PZQ-susceptible strain.

## Materials and methods

### Praziquantel

Praziquantel was purchased from Merck & Co. (Kenilworth, NJ, USA) and dissolved in 1% dimethyl sulfoxide (DMSO) from Sigma-Aldrich as described by Melman et al. (2009), used for stock solution, which was subsequently diluted to appropriate concentrations in culture media.

### Parasite isolation and animal model

In this study we used two parasite strains, a *S. mansoni* BH strain from Belo Horizonte, Minas Gerais, Brazil, susceptible to PZQ, and a stable PZQ-resistant strain (IHMT-LISBON) obtained from the BH strain as described by Pinto-Almeida et al. (2015). Briefly, our stable PZQ-resistant parasite strain was obtained from the PZQ-susceptible BH line submitted to various steps of PZQ continuous drug pressure, starting with 300 mg/kg and finishing with 1200 mg/ kg of PZQ. Infected CD1 mice were checked approximately 60 days post parasite infection by Kato-Katz procedure; if eggs were found in feces, mice were then treated orally with PZQ solution at appropriate dosage. If, on day 15, post PZQ treatment, viable eggs (verified by live miracidia inside the eggs and Kato-Katz procedure) continued to be eliminated, mice were euthanized and miracidia present in the liver were used to subsequently infect *Biomphalaria glabrata* snails. Once *B. glabrata* snails start eliminating *S. mansoni* cercariae (30–60 days after snail infection), new CD1 mice were re-infected and the previous procedure was repeated, continuing the PZQ-resistant strain selection *in vivo*. PZQ dosage was increased every two passages. These two parasite strains are routinely kept in their intermediate host *B. glabrata* snails at our laboratory at Instituto de Higiene e Medicina Tropical, Universidade Nova de Lisboa.

*Mus musculus* CD1 line male mice are considered the choice animal model for *S. mansoni* infection, because it is highly susceptible to this parasite as it closely resembles the *S. mansoni* human infection (Katz and Coelho, 2008). The infection occurred by percutaneous exposure of mice tails to about 100 cercariae of *S. mansoni* each, through natural transdermal penetration of the cercariae (Lewis, 1998).

**Figure 1:**
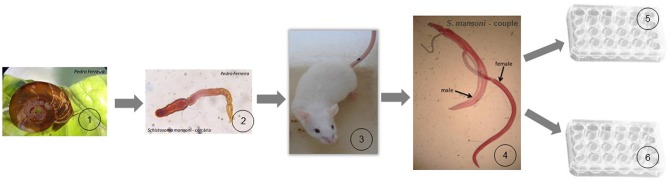
**Schematic cartoon of the experimental design. (1)** *Biomphalaria glabrata* snails (intermediate hosts of *S. mansoni*) release the infective form of the parasite (cercariae) for human or other mammalian definitive hosts; **(2)** about 100 cercariae were used to infect the definitive host; **(3)** CD1 Mice were used as definitive host in our experiment, and after 8–10 weeks post-infection they were sacrificed to collect adult worms of the parasite; **(4)** adult worms were obtained by mice liver perfusion; **(5)** male and female worms were treated in 24-well culture plate with a dosage of PZQ (0.3 μM) with impact in the parasite but with the guarantee of not killing them. These worms were prepared for tegumental alterations study using SEM; **(6)** couple worms were treated in 24-well culture plate with a lethal dosage of PZQ (32 μM). These worms were analyzed and monitored under an inverted optical microscope.

Adult worms (8–10 weeks post-infection) were collected through hepatic portal system and mesenteric veins perfusion, as described by Lewis (1998), washed in saline solution and then maintained in a RPMI medium (Sigma-Aldrich). The Figure 1 explain in more detail the experimental design performed in this study.

### *In vitro* treatment with praziquantel

After collection, adult worm parasites were transferred to 24-well culture plates containing RPMI-1640 culture medium, 200 mM L-glutamine, 10 mM HEPES, 24 mM de NaHCO_3_, 10000 UI of penicillin and 10 mg/mL of streptomycin, from Sigma-Aldrich, pH 7, and supplemented with 15% fetal bovine serum. About five parasites, individually or as a couple, were added to each well and the same concentration of drug was used in two wells. All experiments were carried out in tree biological replicates, 10 on each replicate (*n* = 30) for each studied group: (1) PZQ-susceptible male worms, (2) PZQ-susceptible female worms, (3) PZQ-resistant male worms, (4) PZQ-resistant female worms, (5) PZQ-susceptible couple worms, and (6) PZQ-resistant couple worms. Parasites were incubated overnight at 37°C in a 5% CO_2_ atmosphere to recover from stress caused by perfusion. After this period, male and female worms were treated in culture with a dosage of PZQ with impact in the parasite, but with the guarantee of not killing them (0.3 μM) for 3 h, then washed twice with saline solution to clean any traces of culture medium and prepared for scanning electron microscopy (SEM). Couple worms of both strains were treated *in vitro* with a lethal dosage of PZQ (32 μM) for 48 h. During this period, worms were analyzed and monitored under an inverted optical microscope (DM-500, Leica), to evaluate and monitor the motility and viability of the worms, and morphological changes in eggs of the two parasite strains studied in this work. For negative control of each group, worms were kept in RPMI-1640 drug free medium, under the same conditions.

### Scanning electron microscopy

To evaluate tegumental morphologic changes in both strains of *S. mansoni* after *in vitro* exposure to PZQ, adult worms were analyzed using scanning electron microscopy as described in de Oliveira et al. (2012, 2013, 2014). Briefly, worms were incubated at 37°C in a CO_2_ atmosphere (5%) for 24 h. After incubation, the parasites were washed with 0.1 M sodium cacodylate buffer (for 1 h, changing the solution every 15 min), fixed in 2.5% glutaraldehyde (pH 7.4) (Merck) for 24 h and then fixed in 1% osmium tetroxide for 1 h. Specimens were dehydrated in increasing concentrations of ethanol (50, 70, 80, 90, 95, and 100%) for 30 min each, dried in a critical point dryer, mounted on stubs, metalized with gold particles using Sputter Coater and finally analyzed and photographed using an ultra-scanning electron microscope (Jeol-JSM-820).

### Ethics statement

Animal housing was maintained at 25 ± 2°C and 45–65% relative humidity. All the animal experimental were approved by the Ethics and Animal Welfare Committee (CEBEA), Faculty of Veterinary Medicine, UTL (Ref. 0421/2013) and performed according to the committee guidelines. Animals were kept and handled in accordance with National and European legislation (DL 276/2001 and DL 314/2003; 2010/63/EU adopted on 22nd September 2010), with regard to animal protection and welfare, and all procedures were performed according to National and European legislation. The anesthetics and other techniques were used to reduce the pain and adverse effect of animal.

### Statistical analysis

Results were expressed as mean ± SD. Data was statistically analyzed using the IBM SPSS Statistics software version 19.0 for Windows. Kolmogorov-Smirnov (Lilliefors significance correction) and Shapiro-Wilk tests were used to analyse data normality and Levene's test was used to test homogeneity of variance. After this we chose to use a parametric test—*t*-test for independent samples, to compare the average size of the eggs and lateral spines between PZQ-resistant and PZQ-susceptible strains. On the other hand, due to the lack of normality of the data and lack of homogeneity of variance, we used a non-parametric test—Mann–Whitney (MW), to test whether there was difference between the ratios of lateral spines/eggs on the two strains studied. The level of significance was set at *p* < 0.05.

## Results

### *In vitro* effect of PZQ on *S. mansoni* PZQ-resistant and -susceptible strains

**Figure 2:**
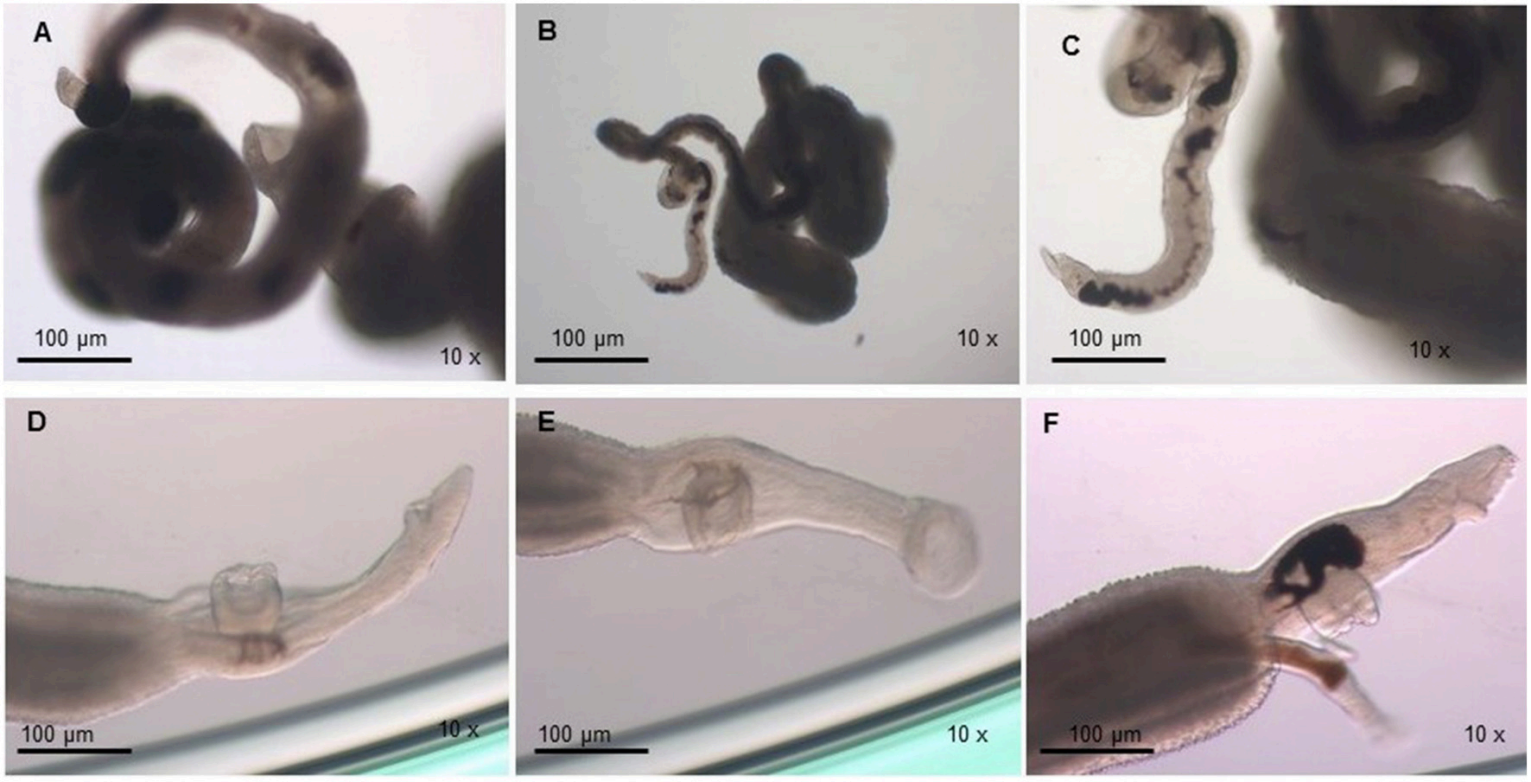
**Monitoring of *S. mansoni* resistant strain adult worms submitted to 32 μM of PZQ during 48 h. (A)** Adult worms from the resistant strain exposed to PZQ, showing muscle contraction, and reduction of movements; **(B)** Adult worms from the resistant strain exposed to PZQ, showing muscle contraction, and little movements (24 h after drug exposure); **(C)** Adult worms from the resistant strain exposed to PZQ, began to gain some motility by the end of the incubation period (48 h); **(D)** Adult worms from the resistant strain not exposed to PZQ (negative control group—resistant worms kept in RPMI-1640 medium with no addition of the drug); **(E)** Adult worms from the resistant strain not exposed to PZQ (negative control group—resistant worms kept in RPMI-1640 medium with no addition of the drug), 24 h of incubation period; **(F)** Adult worms from the resistant strain not exposed to PZQ (negative control group—resistant worms kept in RPMI-1640 medium with no addition of the drug), at the end of the incubation period (48 h).

The viability of the adult worms was analyzed during *in vitro* incubation with a concentration of 32 μM of PZQ for a 48 h period. We observed that the viability of adult worms resistant to PZQ and treated with this drug was similar to the negative resistant control group (resistant worms kept in RPMI-1640 drug free medium), in which all individuals were alive after the incubation period. We noticed that upon exposure to PZQ adult worms from the resistant strain retracted, showed muscle contraction, reduction of movements (Figures 2A,B) and, by the end of the incubation period, start regaining motility (Figure 2C), in comparison to the control group (Figures 2D–F). Still, the worms remained alive and, after removal of the medium containing PZQ, they gradually regained full motility.

**Figure 3:**
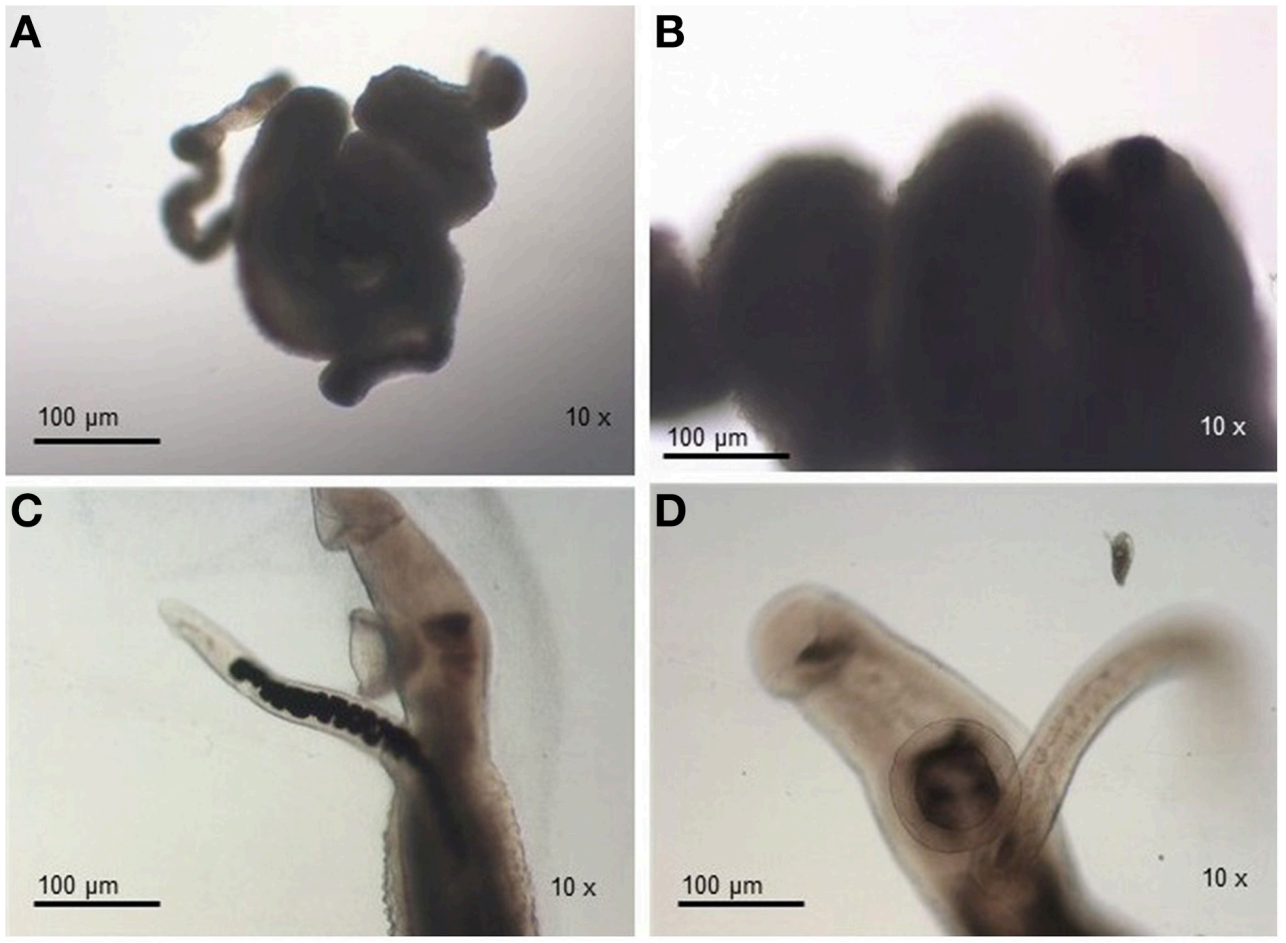
**Monitoring of *S. mansoni* susceptible strain adult worms submitted to 32 μM of PZQ during 48 h. (A)** Adult worms from the susceptible strain exposed to PZQ, showing muscle contraction and reduction of movements; **(B)** Adult worms from the susceptible strain dead after exposed to PZQ, 24 h after exposure; **(C)** Adult worms from the susceptible strain not exposed to PZQ (negative control group—susceptible worms kept in RPMI-1640 medium with no addition of the drug); **(D)** Adult worms from the susceptible strain not exposed to PZQ (negative control group—susceptible worms kept in RPMI-1640 medium with no addition of the drug), 24 h of incubation period.

For susceptible parasites, where most of the worms were dead after drug addition, the viability was much lower compared to the negative susceptible control group (susceptible worms kept in RPMI-1640 drug free medium) and the treated-resistant parasite group. Susceptible strain parasites were more contracted (Figure 3A) than those from the resistant isolate submitted to the same PZQ dosage (Figures 2A,B). After exposure to PZQ, drug-susceptible adult worms retracted, showed muscle contraction, and reduction of movements (Figure 3A), in comparison to the control group (Figures 3C,D), and dead after 24 h of incubation period (Figure 3B). After removal of the medium containing PZQ, they did not recover motility, contrary to what happened with resistant strain parasites that gradually regained motility.

**Figure 4:**
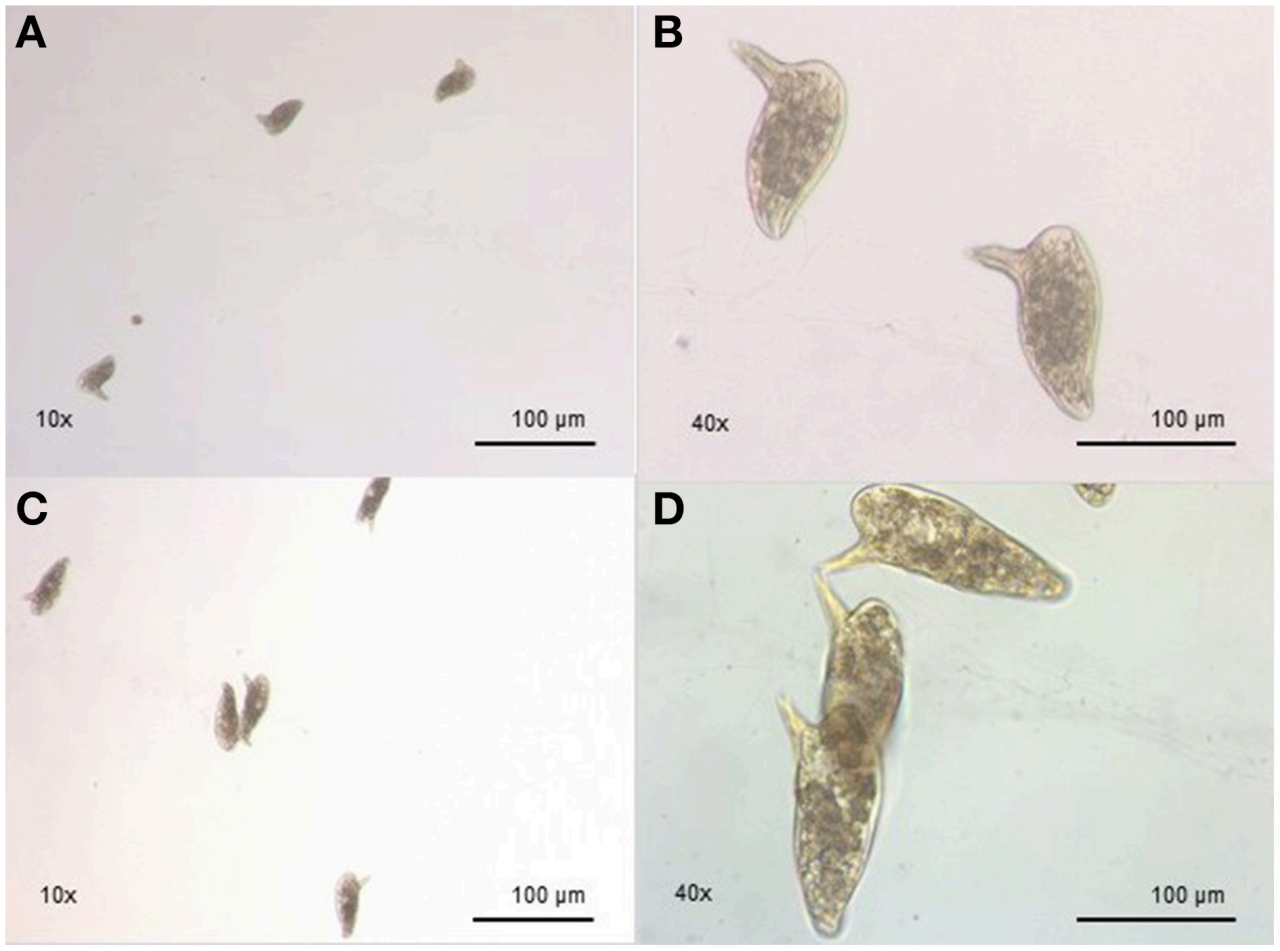
**Morphological difference between eggs from resistant strain and susceptible strain. (A)** Eggs from resistant strain parasites, showing morphology alterations, smaller size, and smaller lateral spines (10x); **(B)** Eggs from resistant strain, in a bigger scale, showing morphology alterations, smaller size, and smaller lateral spines (40x); **(C)** Eggs from susceptible strain parasites, showing normal morphology (10x); **(D)** Eggs from susceptible strain parasites, showing normal morphology, in a bigger scale (40x).

**Figure 5:**
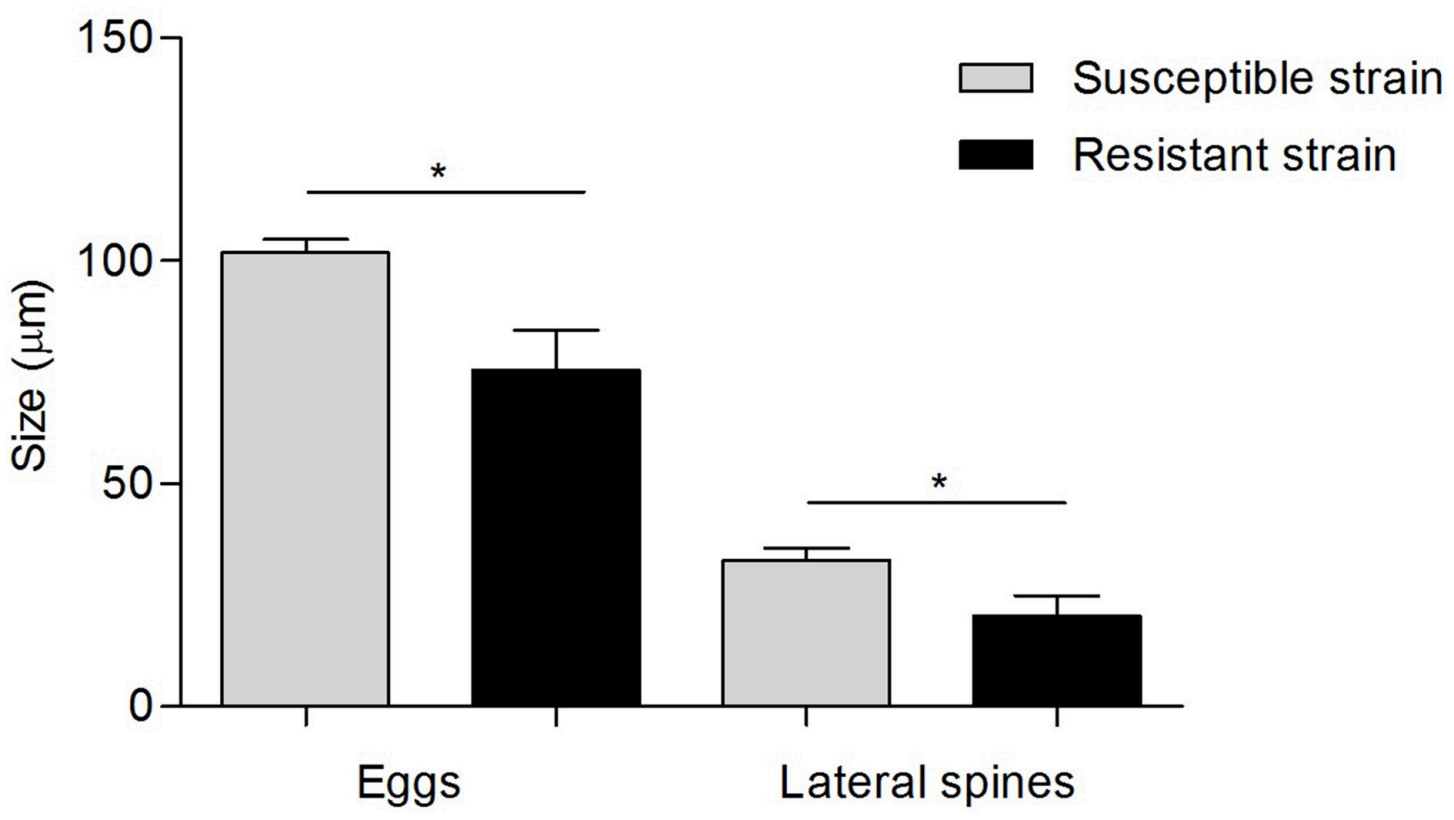
**Difference in egg morphology of *S. mansoni* resistant strain and susceptible strain** Gray bars, measurements of eggs and lateral spines from susceptible strain worms; Black bars, measurements of eggs and lateral spines from resistant strain worms. Data was presented as mean ± SD. Statistical analysis was performed by parametric *t*-test, for independent simples, whose level of significance was set at *p* < 0.05. ^*^ indicated *p* < 0.05.

Regarding the egg morphology, it was different between PZQ-resistant and PZQ-susceptible stains (Figure 4). Eggs from resistant parasite females (Figures 4A,B, *n* = 7) were smaller (*p* < 0.05) when compared to those from the susceptible strain (Figures 4C,D, *n* = 7; Figure 5 and Table 1). Furthermore, we found statistical significant differences in the size of the lateral spines of the eggs, those from resistant strain females had smaller and thicker lateral spines than those of susceptible worms (*p* < 0.05; Figure 5 and Table 1). In order to confirm that the observed differences in lateral spine size were not a simple consequence of smaller size of the egg in resistant worms, the ratio between lateral spine and egg sizes was determined for resistant strain and susceptible strain (Table 1). When the ratios (lateral spine size/egg size) of the two strains were compared, we found a significant difference (*p* < 0.05) between eggs from resistant worms and susceptible worms, which mean that the difference observed in the size of the lateral spine is not a consequence of smaller size of the eggs. These are interesting findings since it may have important repercussions on pathological and symptomatology effects induced by this strain.

**Table 1:** **Difference in egg morphology (size of the eggs and lateral spine and ratio between them) of *S. mansoni* resistant and susceptible parasites (*n* = 7)**.

**Egg morphology**	**Size (μm) mean ± SD**	**95% Confidence limits**	***p*-value**
Susceptible strain eggs	101.80 ± 2.99	99.03–104.57	*p* < 0.05 (*t*-test)
Resistant strain eggs	75.60 ± 8.98	67.29–83.91	
Susceptible strain lateral spines	32.78 ± 2.75	30.24–35.33	*p* < 0.05 (*t*-test)
Resistant strain lateral spines	20.26 ± 4.73	15.88–24.64	
Susceptible strain ratio (SS spine/SS egg)	0.32 ± 0.018	0.30–0.34	*p* < 0.05 (MW test)
Resistant strain ratio (RS spine/RS egg)	0.27 ± 0.036	0.23–0.29	

*SS, susceptible strain; RS, resistant strain*.

### Effect of PZQ on tegument of *S. mansoni* PZQ-resistant and -susceptible strains

**Figure 6:**
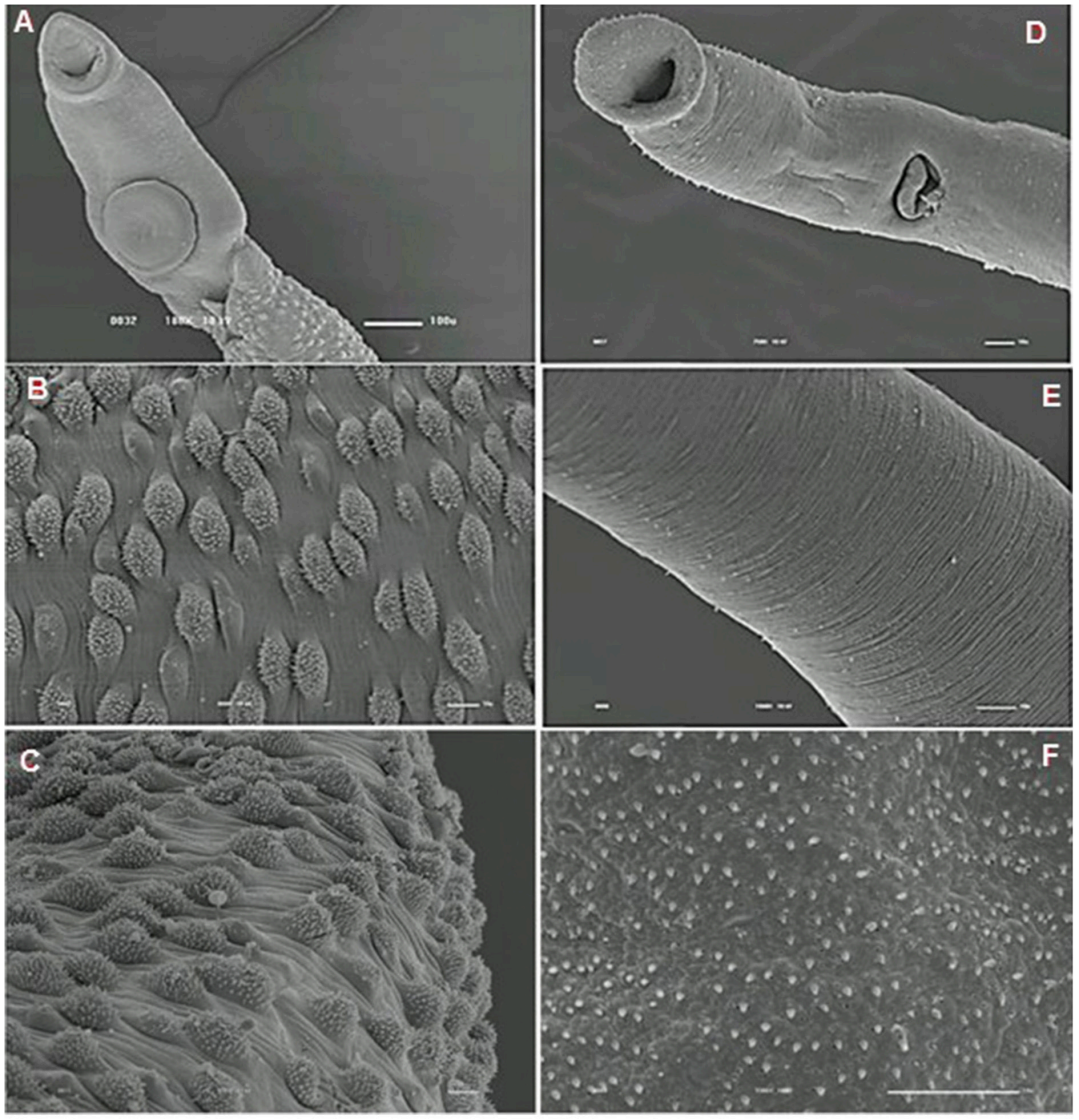
**Scanning electron microscopy of *S. mansoni* PZQ-susceptible strain**. **(A–C)** Susceptible strain adult males of control group kept in RPMI-1640 drug free medium for 3 h, showing normal morphology of the tegument, and oral and ventral suckers; **(D–F)** Susceptible strain adult females of control group kept in RPMI-1640 drug free medium for 3 h, showing normal morphology of the tegument, and oral and ventral suckers. Image marginations: **(A)** 180x 10 kV ________100u; **(B)** 900x 10 kV ________10u; **(C)** 800x 10 kV ________10u; **(D)** 500x 10 kV ________10u; **(E)** 1200x 10 kV ________10u; **(F)** 3300x 10 kV ________10u.

**Figure 7:**
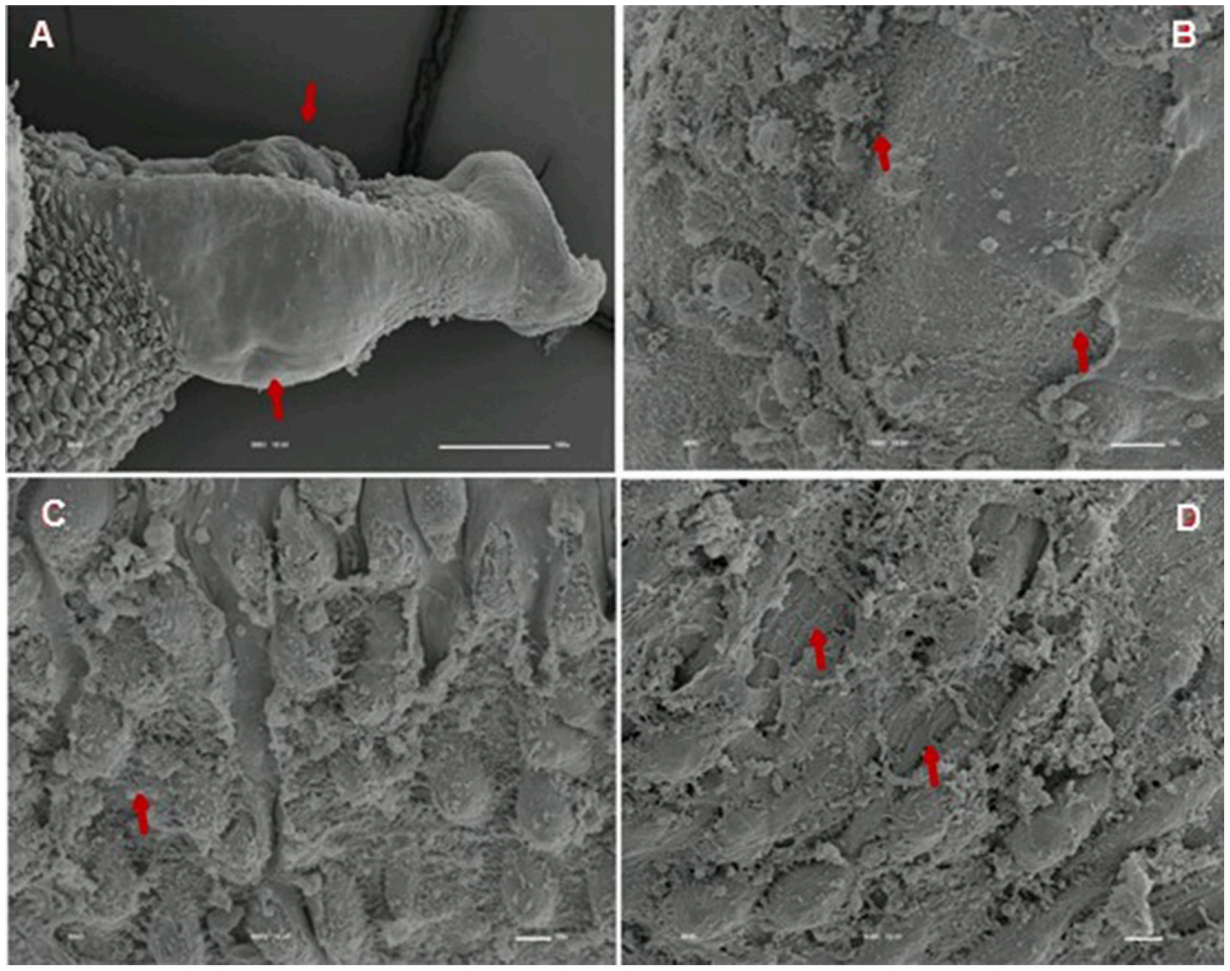
**Scanning electron microscopy of *S. mansoni* PZQ-susceptible strain adult males after exposure to 0.3 μM of PZQ for 3 h**. **(A)** Susceptible strain adult males upon exposure to PZQ, presenting changes in acetabular suckers; **(B)** tegument peeling; **(C,D)** destruction of tubercles and spines. Red arrows indicate alterations. Image marginations: **(A)** 300x 10 kV ________100u; **(B)** 1200x 10 kV ________10u; **(C)** 800x 10 kV ________10u; **(D)** 850x 10 kV ________10u.

**Figure 8:**
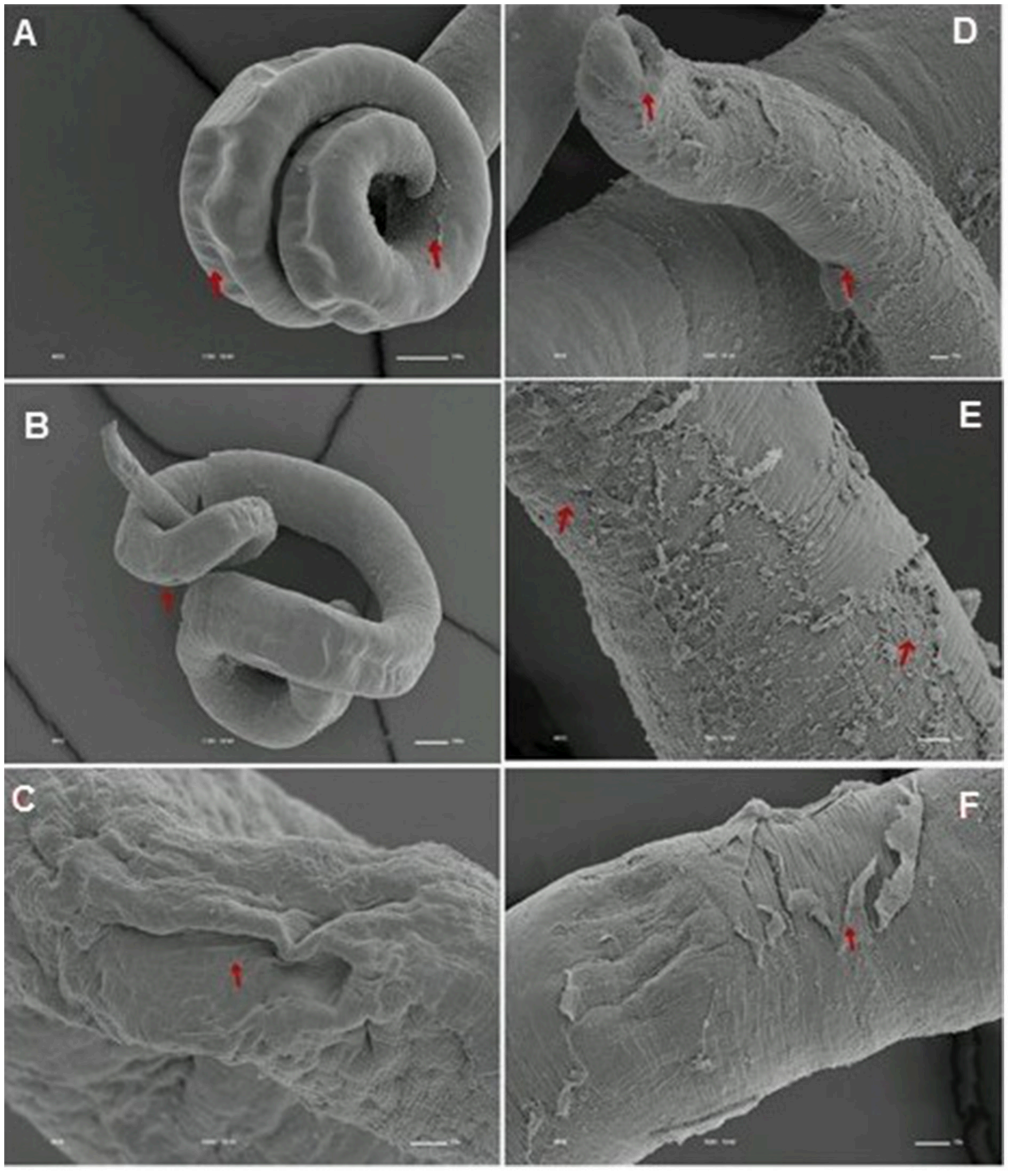
**Scanning electron microscopy of *S. mansoni* PZQ-susceptible strain adult females after exposure to 0.3 μM of PZQ for 3 h**. **(A–C)** Susceptible strain adult females upon exposure to PZQ, showing muscle contraction and corrugations; **(D)** alterations in oral sucker; **(E,F)** peeling of some tegumental regions. Red arrows indicate alterations. Image marginations: **(A)** 170x 10 kV ________100u; **(B)** 110x 10 kV ________100u; **(C)** 1000x 10 kV ________10u; **(D)** 500x 10 kV ________10u; **(E)** 900x 10 kV ________10u; **(F)** 950x 10 kV ________10u.

We analyzed the presence of tegumental alterations in *S. mansoni* PZQ-resistant strain adult worms and in the parental PZQ-susceptible strain upon addition of 0.3 μM PZQ during 3 h, using scanning electron microscopy. Significant changes were only observed in males and female worms of the susceptible strain. In susceptible males not exposed to PZQ, the oral and ventral sucker (Figure 6A) and tegument (Figures 6B,C) did not show any changes, while males exposed to the same drug presented changes in acetabular sucker (Figure 7A), tegument peeling (Figure 7B), and destruction of tubercles and spines (Figures 7C,D). Susceptible strain females not exposed to PZQ showed normal morphology of ventral and oral suckers (Figure 6D) and normal morphology of the tegument (Figures 6E,F), but when exposed to the drug, presented muscle contraction and corrugations (Figures 8A–C). Furthermore, they showed alterations in the oral sucker (Figure 8D) and peeling of some tegumental regions (Figures 8E,F).

**Figure 9:**
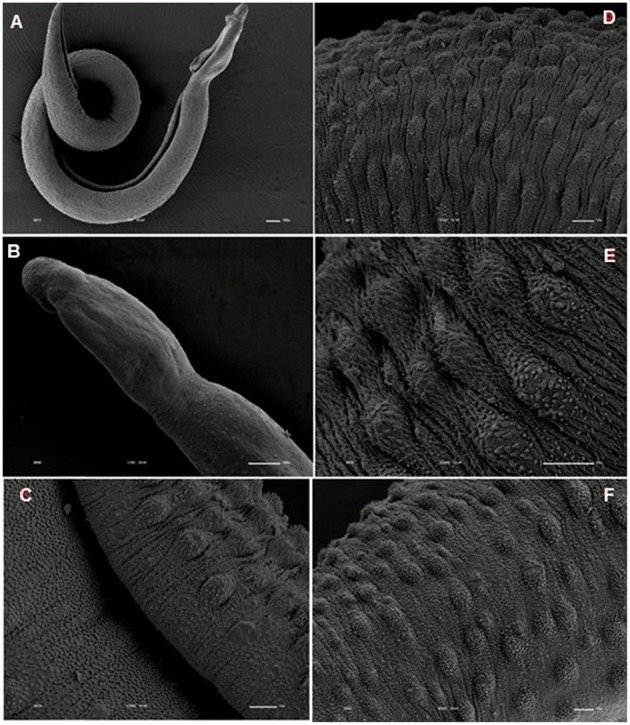
**Scanning electron microscopy of *S. mansoni* PZQ-resistant strain adult males of control group kept in RPMI-1640 drug free medium for 3 h**. **(A,B)** Resistant strain adult males kept in RPMI-1640 drug free medium, showing normal morphology of the oral and ventral suckers; **(C–F)** normal morphology of the tegument. Image marginations: **(A)** 70x 10 kV ________100u; **(B)** 170x 10 kV ________100u; **(C)** 1200x 10 kV ________10u; **(D)** 950x 10 kV ________10u; **(E)** 2200x 10 kV ________10u; **(F)** 850x 10 kV ________10u.

**Figure 10:**
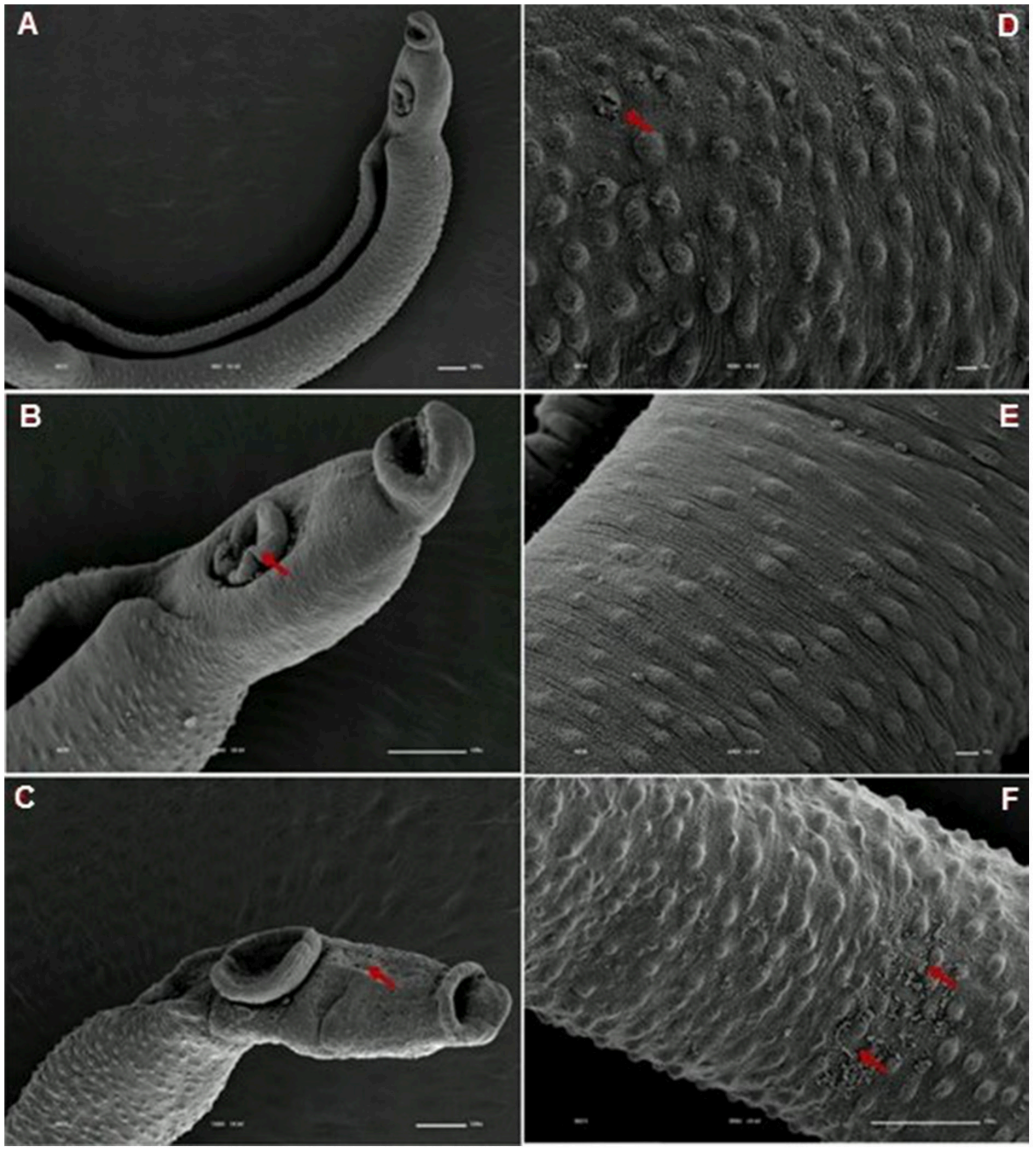
**Scanning electron microscopy of *S. mansoni* PZQ-resistant strain adult males after exposure to 0.3 μM of PZQ for 3 h**. **(A–C)** Resistant strain adult males upon exposure to PZQ, presenting changes in oral and ventral suckers; **(D–F)** losses of tubercles and spines. Red arrows indicate alterations. Image marginations: **(A)** 90x 10 kV ________100u; **(B)** 250x 10 kV ________100u; **(C)** 160x 10 kV ________100u; **(D)** 550x 10 kV ________10u; **(E)** 600x 10 kV ________10u; **(F)** 350x 10 kV ________100u.

**Figure 11:**
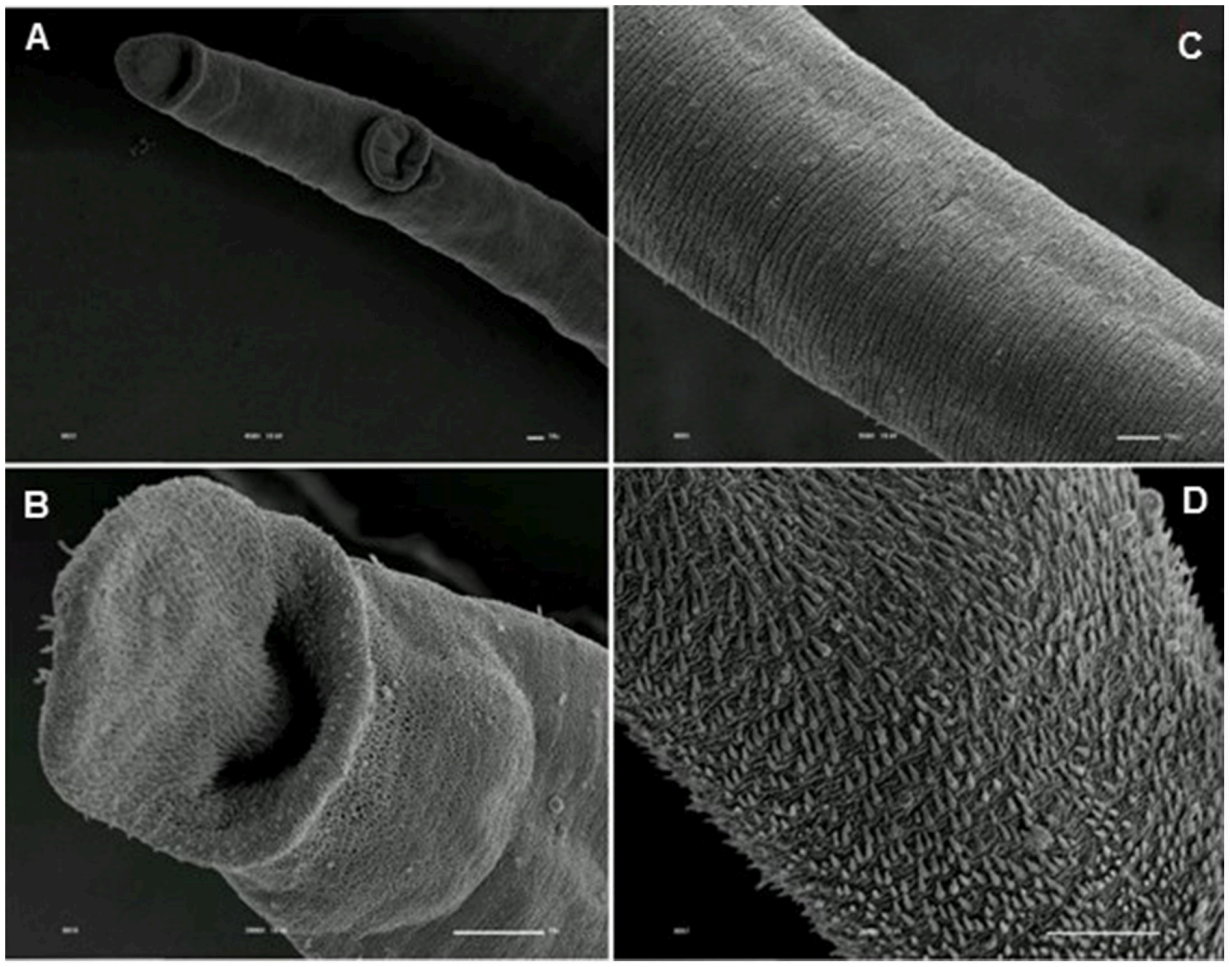
**Scanning electron microscopy of *S. mansoni* PZQ-resistant strain adult females of control group kept in RPMI-1640 drug free medium for 3 h**. **(A,B)** Resistant strain adult females kept in RPMI-1640 drug free medium, showing normal morphology of the oral and ventral suckers; **(C,D)** normal morphology of the tegument. Image marginations: **(A)** 450x 10 kV ________10u; **(B)** 2000x 10 kV ________10u; **(C)** 950x 10 kV ________10u; **(D)** 2500x 10 kV ________10u.

**Figure 12:**
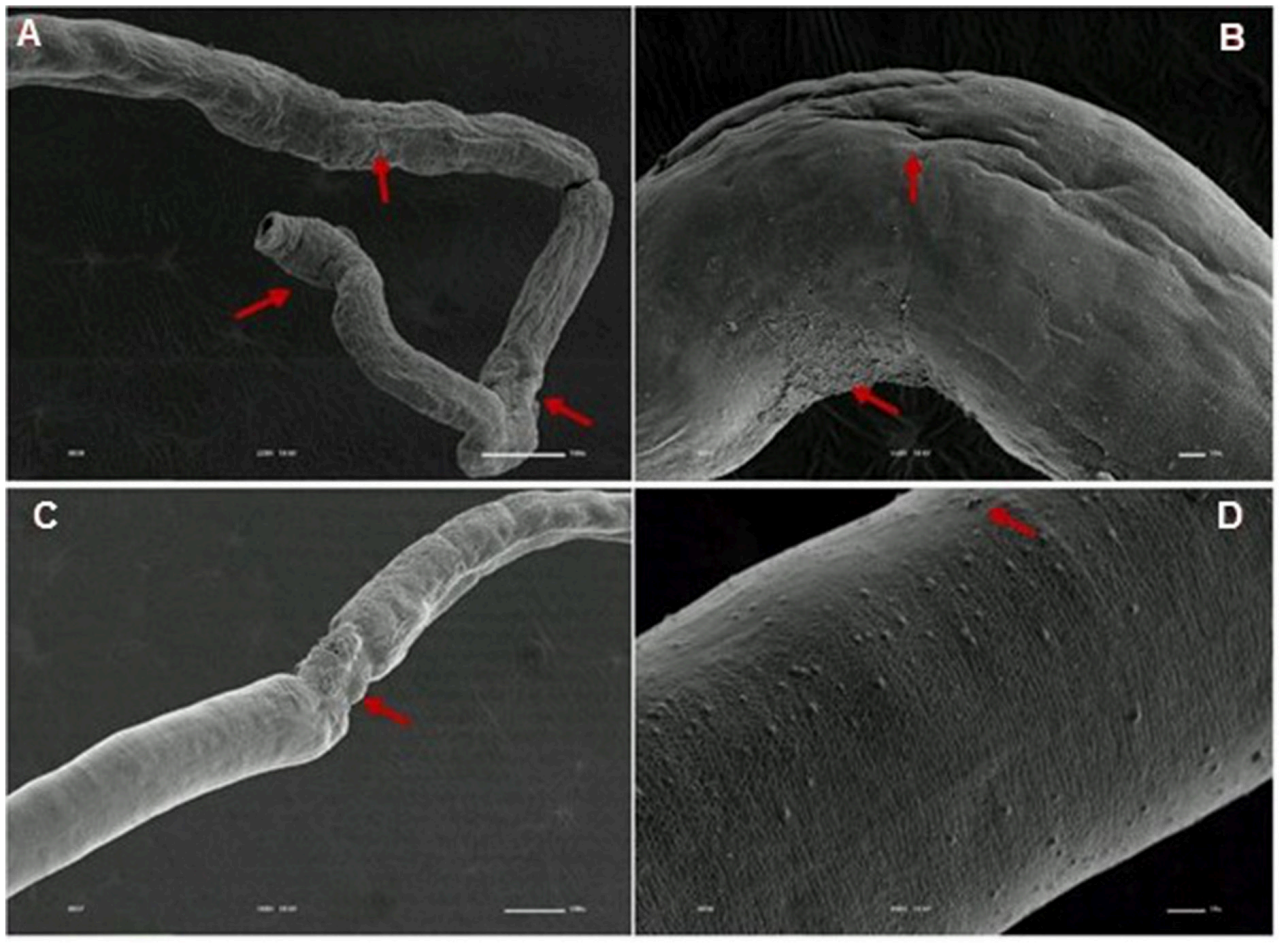
**Scanning electron microscopy of *S. mansoni* PZQ-resistant strain adult females after exposure to 0.3 μM of PZQ for 3 h**. **(A)** Resistant strain adult females upon exposure to PZQ, presenting changes in oral suckers; **(B)** light peeling in the worm ventral region; **(C,D)** alterations in some tegumental areas. Red arrows indicate alterations. Image marginations: **(A)** 220x 10 kV ________100u; **(B)** 550x 10 kV ________10u; **(C)** 160x 10 kV ________100u; **(D)** 900x 10 kV ________10u.

Tegumental alterations induced by PZQ were not so significant in the resistant strain, when compared to the susceptible strain. As expected, the control group of resistant males did not show any tegumental alterations, showing normal morphology of the oral and ventral suckers (Figures 9A,B) and tegument (Figures 9C–F). When exposed to PZQ, those worms presented small alterations in some areas, such as changes in oral and ventral suckers (Figures 10A–C), and little alterations in the body surface, with losses of tubercles and spines (Figures 10D–F). Similarly female resistant worms control group did not present any tegumental damages, neither in oral and ventral suckers (Figures 11A,B), nor in the tegument (Figures 11C,D). Upon drug exposure, those worms only showed very few alterations, namely, some morphological changes in the oral sucker (Figure 12A), some light peeling in the worm ventral region (Figure 12B), and alterations in some tegumental areas (Figures 12C,D).

## Discussion

In past years, several studies have been performed in order to demonstrate that resistance/tolerance to PZQ may occur and is more than hypothetical (Fallon and Doenhoff, 1994; Ismail et al., 1996; Doenhoff et al., 2002; Cioli et al., 2004). Our group in particular had selected, by stepwise drug pressure, a *S. mansoni* strain that is isogenic to its parental fully susceptible counterpart, except for genetic determinants accounting for the PZQ-drug resistance phenotype, and phenotypically similar to the susceptible strain except in resistance. In the present study, we took the advantage of the availability of these two strains of *S. mansoni* and performed a comparative assessment of morphological alterations that the *in vitro* effect of PZQ can cause on *S. mansoni* PZQ-resistant parasites. William et al. (2001) and Liang et al. (2001) have already performed studies with PZQ-resistant isolates obtained from an Egyptian and a Senegalese patient eggs, which were not cured by three therapeutic doses of PZQ, where they demonstrated that isolates from resistant infections were less susceptible to Praziquantel-induced tegumental damage *in vitro* (William et al., 2001) and Praziquantel-resistant isolates may be more pathogenic in mice than the susceptible ones (Liang et al., 2001). However, as far as we know, our study is the first report in which the *in vitro* effect of PZQ on the morphological characteristics of a resistant strain of *S. mansoni* can be compared with its parental susceptible strain.

Parameters, such as motor activity, eggs morphology, and tegumental changes, are often evaluated as indicators of biological activity in studies using schistosomes species (William et al., 2001; Sanderson et al., 2002; Pica-Mattoccia and Cioli, 2004; De Araújo et al., 2007; Xiao et al., 2007; de Oliveira Penido et al., 2008; Katz, 2008; Boissier et al., 2009; Magalhães et al., 2009, 2010), hence we evaluate these parameters in order to assess the effect of PZQ on adult *S. mansoni* PZQ-resistant and PZQ-susceptible worms survival and fitness.

Our study shows that resistant strain worms have less muscle contraction and movements after exposure to PZQ than susceptible isolates. This complies with studies suggesting that contraction of somatic musculature is a marked effect of addition of PZQ to schistosomes *in vitro* (William et al., 2001), and that worms resistant to PZQ *in vivo* have significantly reduced contractile responses to PZQ *in vitro* (Ismail et al., 1996). It is also evident that, after removal of the medium containing the drug, resistant worms recover motility, unlike susceptible worms, where the majority of which are dead.

The tegument is a very important organ for schistosomes, for many reasons: it is important for the survival of the worms in the host (Skelly and Wilson, 2006; Van Hellemon et al., 2006; Moraes, 2012), protecting the parasite against the action of the host's immune system, absorbing nutrients and molecules and participating in synthesis of some proteins (Shuhua et al., 2000; Bertão et al., 2012; Reda et al., 2012). Some studies carried out many years ago, tried to clarify the action mechanisms of the drug used in schistosomiasis treatment. It was shown that, worms subjected to PZQ, have vacuolization of the tegument and disruption of the apical tegumental layer (Becker et al., 1980). Female worms present tegumental damage, such as, tegument and sub-tegument vacuolization and tegument and musculature destruction, while male worms show more pronounced and extensive surface alterations, which include surface bleeding, swellings, wrinkling, constrictions, and surface lesions, particularly on the spined tubercles (Shaw and Erasmus, 1983).

As shown by William et al. (2001), our findings demonstrate that tegumental damage caused by the *in vitro* effect of PZQ is much less evident in resistant strain adult worms than in the susceptible strain. Contrary to what occurs in resistant parasites, in which PZQ does not seem to cause major damage, males of the susceptible strain present tegument peeling, tubercle and spine destruction and vesicles around the tubercles while females display peeling and wrinkling of the tegument and destruction of oral and acetabular suckers. These suggest that there might be a difference in the resistant strain tegument composition that may render the worms less responsiveness to PZQ. Therefore, it will be very interesting to perform a more in-depth study of the resistant strain tegument to see if PZQ resistance might in any way influence pathology symptomology, since one of the hallmark effects of PZQ on schistosomes *in vitro* is the disruption of surface tegument (William et al., 2001). It is important to notice that susceptible strain females presented much less damage than males. This observation is in agreement to the findings from our previous study where females appeared more tolerant to PZQ (Pinto-Almeida et al., 2015). In the resistant strain, damages on both males and females were so small that it is far more difficult to make a similar comparison.

It has been stated in literature that the oviposition of *S. mansoni* during *in vitro* culture of adult worms show three very distinct phases in the kinetics of oviposition: an initial phase with low egg production, a period of maximum oviposition and finally a gradual reduction in the number of eggs during the last phases of culture (Barth et al., 1996). Liang et al. (2001) demonstrated that mice infected with PZQ-resistant isolates shed more eggs in their feces than those carrying drug-susceptible parasites and mice infected with any of the resistant isolates also had larger numbers of eggs in their tissues. Mice infected with our PZQ-resistant strain shed more eggs than those infected by the counterpart susceptible strain. This is important because if there is a change in the biological characteristics of schistosomes associated with the development of resistance to PZQ, it could affect the transmission and pathology of the diseases they cause (Liang et al., 2001).

Another interesting finding observed in this study is that resistant strain eggs were smaller and had a smaller lateral spine in comparison with eggs from susceptible strain, which may have repercussions in the pathology of the disease. We have observed that some mice infected with the PZQ-resistant strain used here presented some neurological manifestations, including deviation of the head, tendency to roll over on stimulation, ataxia, and convulsions, very similar to what is usually seen in mice affected with cerebral malaria (Lou et al., 2001). Based on this, it is not unreasonable to speculate that resistant strain parasites might have altered tissue tropism, namely, a higher tropism for brain or spinal cord, which may potentiate the development of neurological manifestations. Clearly, further studies on mice infected with PZQ-resistant isolates are required to confirm this hypothesis.

In conclusion, we compared morphological characteristics of *S. mansoni* PZQ-resistant and PZQ-susceptible strains upon addition of this drug *in vitro*. It was demonstrated that the resistant strain presents (i) less muscular contractions, (ii) less tegumental damage, (iii) more viability, and (iv) recovering motility when the drug is removed, indicating fully active life after a PZQ treatment is ceased. The resistant strain demonstrated different egg morphology when compared with susceptible strain. Those are important findings since any biological changes can produce relevant alterations in the transmission and pathology of diseases. Comparing two strains that only differ in resistance characteristics is an important step in the study of schistosomiasis as it guarantees that the differences observed between the two strains are closely related to resistance. Increase tolerance/resistance to PZQ in an *in vivo* environment is an obvious fact and studies should be performed to clarify the mechanisms associated with it. This study certainly opens doors for further in-depth *S. mansoni* drug resistance studies.

## Author contributions

AP, TM, RD, SD—Have done all the experimental work, experimental results treatment and manuscript writing. SA, SB, AT, FD, EC, AA—Have designed all experiments and done experimental results treatment and manuscript writing. EC, AA—Are responsible for financial questions.

### Conflict of interest statement

The authors declare that the research was conducted in the absence of any commercial or financial relationships that could be construed as a potential conflict of interest.
